# Underwater Docking Approach and Homing to Enable Persistent Operation

**DOI:** 10.3389/frobt.2021.621755

**Published:** 2021-03-15

**Authors:** Brian R. Page, Reeve Lambert, Jalil Chavez‐Galaviz, Nina Mahmoudian

**Affiliations:** School of Mechanical Engineering, Purdue University, West Lafayette, IN, United States

**Keywords:** marine robot navigation, autonomous underwater docking, underwater docking technique, marine robotics, underwater robot, autonomous underwater vehicle

## Abstract

One of the main limiting factors in deployment of marine robots is the issue of energy sustainability. This is particularly challenging for traditional propeller-driven autonomous underwater vehicles which operate using energy intensive thrusters. One emerging technology to enable persistent performance is the use of autonomous recharging and retasking through underwater docking stations. This paper presents an integrated navigational algorithm to facilitate reliable underwater docking of autonomous underwater vehicles. Specifically, the algorithm dynamically re-plans Dubins paths to create an efficient trajectory from the current vehicle position through approach into terminal homing. The path is followed using integral line of sight control until handoff to the terminal homing method. A light tracking algorithm drives the vehicle from the handoff location into the dock. In experimental testing using an Oceanserver Iver3 and Bluefin SandShark, the approach phase reached the target handoff within 2 m in 48 of 48 tests. The terminal homing phase was capable of handling up to 5 m offsets with approximately 70% accuracy (12 of 17 tests). In the event of failed docking, a Dubins path is generated to efficiently drive the vehicle to re-attempt docking. The vehicle should be able to successfully dock in the majority of foreseeable scenarios when re-attempts are considered. This method, when combined with recent work on docking station design, intelligent cooperative path planning, underwater communication, and underwater power transfer, will enable true persistent undersea operation in the extremely dynamic ocean environment.

## 1 Introduction

Understanding the marine environment is critical for a variety of missions ranging from safety and surveillance to biological studies and global weather forecasting. Despite this, long-term monitoring is currently limited to a few unique platforms such as Waveglider (Hine et al., 2009) and Slocum (Webb et al., 2001). This is largely due to the energy sustainability issue.

Existing persistent-autonomous platforms can be generally broken down into two categories: 1) underwater gliders and 2) surface energy harvesters. While both categories provide persistent presence in oceanic applications, they suffer from limitations in movement freedom and speed.

Underwater gliders locomote through controlling their net buoyancy. The net buoyant force causes the glider to sink (or rise) and an external wing along with internal actuation translates the purely vertical motion into sawtooth or helical flight (Zhang et al., 2013). This motion is incredibly efficient, as the vehicle only needs to expend significant energy during changes in net buoyancy. This results in vehicle endurance on the order of months to years (Jones, 2012), especially when thermal harvesting is included (Ma et al., 2016). The two primary drawbacks to glider deployments are caused by limitations imposed by their buoyancy driven nature. As gliders operate by converting vertical velocity into forward velocity with an external wing, they are unable to travel in level trajectories. Additionally, their operational speeds are generally very slow (on the order of one knot (Page et al., 2017)), and turning radiuses are on the order of 30–50 m (Sherman et al., 2001). These limitations preclude their use on mapping, survey, and surveillance missions.

The other broad category of persistent platforms is surface energy harvesters such as the Waveglider (Hine et al., 2009). These platforms operate by converting energy from the environment such as wave, wind, or solar into locomotive power. They are highly capable platforms and have been widely used on long duration missions approaching true persistence (Manley and Willcox, 2010; Wiggins et al., 2010). However, these vehicles must remain on the surface and are unable to survey the ocean environment at depth.

Due to the restrictions imposed by these alternate locomotion methods, the vast majority of underwater missions are completed using traditional Autonomous Underwater Vehicles (AUVs). These vehicles generally have a single rear thruster and a set of control surfaces. A wide range of such platforms exist, from a small scale such as the Bluefin SandShark (Naglak et al., 2018) to the large scale MERLIN (Lewis et al., 2016). The majority of AUVs are optimized for operational runtimes on the order of 12–24 h with manual charging and re-tasking required between missions (Page and Mahmoudian, 2020). Unfortunately, this manual charging process interrupts operation and necessitates a manned surface presence significantly increasing cost and risk of any long duration open ocean missions.

One emerging technology to alleviate endurance limitations is recharging and data transfer for AUVs with underwater docking stations. Docking stations come in a range of shapes, sizes, and costs from the more common funnel shaped designs (Brighenti et al., 1998; Allen et al., 2006; McEwen et al., 2008) to vertical docking poles (Singh et al., 2001; Lambiotte et al., 2002). A recent docking station design is the adaptive docking system (Page and Mahmoudian, 2020). Overall, underwater docking systems are highly capable of supporting persistent missions in the oceanic environment as long as the vehicle is able to navigate to and rendezvous with the station.

When operating in the dynamic ocean environment, successful docking is critical for overall mission success. However, no docking algorithm can guarantee a 100% docking rate (Bellingham, 2016) due to disturbances such as slowly varying currents. The goal then becomes to develop an algorithm that is capable of efficiently driving the vehicle towards a docking location with a reasonably high success percentage. The algorithm then must be able to efficiently handle the inevitable failed docking attempts through an intelligent go-around maneuver.

The contribution of this paper is an underwater docking navigation algorithm capable of driving the AUV from the field through approach, terminal homing, and into any funnel shaped docking station. This method utilizes a Dubins re-planning approach and integral line of sight to be able to efficiently navigate towards a terminal homing control handoff location. After handoff, an optical navigation method is able to accurately converge and eventually dock with the docking station. Further, the proposed method is able to efficiently handle the go-around maneuver to manage failed docking attempts. To the best of the authors knowledge, this is the first reported implementation of integral line of sight control for tracking of a dynamic re-planned Dubins path, particularly when considering the underwater docking application.

The proposed algorithm has been validated in two stages on an OceanServer Iver3 and a Bluefin SandShark. Approach navigation was tested using an OceanServer Iver3 due to its use by operators in the field, while terminal homing was tested on a Bluefin SandShark due to its small size. In approach testing, the OceanServer Iver3 successfully navigated to within 1 m of the target handoff point in 39 out of 48 attempts and within 2 m in 48 out of 48 attempts. Further, the method demonstrated efficient re-planning to re-attempt docking. This level of accuracy, when combined with the 70% accuracy (12 of 17 attempts) terminal homing approach validated on a Bluefin SandShark will enable real world underwater persistent operation. For this method, we assume operation in normal oceanic conditions (slowly varying, large scale currents with max flow significantly slower than vehicle speed (Gross, 1977)) with no obstacles, and 2D planar operation. The proposed method is capable of handling both intermittent localization updates as well as continuous environmental disturbances. Intermittent localization updates such as through acoustic tracking or GPS surfacing trigger the path to be re-planned while environmental disturbances are approximated through the integral action of the integral line of sight controller.

The remainder of this paper discusses background in Section 2, algorithm design in Section 3, experimental results in Section 4, and a conclusion/future work in Section 5.

## 2 Background

Docking stations have typically been of either the funnel (Stokey et al., 2001; McEwen et al., 2008) or pole (Singh et al., 2001) design. Power transfer is completed using either inductive power (Stielau and Covic, 2000; Zhang et al., 2014) or stab connections (Bradley et al., 2001; Painter and Flynn, 2006). Inductive power systems are able to transfer power through moderate misalignment and for extremely high cycle counts due to their wireless nature. Existing stations are able to support charging of AUVs to enable persistent operation, yet, they have not seen widespread adoption.

Limiting factors in docking station deployments, in addition to high costs, are the lack of flexibility to support different types of AUVs and the challenge of developing robust control for the AUV docking maneuver. Existing docking stations are installed on the seafloor (Hobson et al., 2007; Bellingham, 2016; Matsuda et al., 2019) or hauled by significantly larger marine assets, such as the *Proteus* (Pyle et al., 2012). An additional option is to mount the dock onto a surface platform to aid launch and recovery (Pearson et al., 2014; Meng et al., 2018; Meng et al., 2019). Recent work has focused on flexible docking and recovery stations that can be towed by small Unmanned Surface Vehicles (USV) (Meng et al., 2018; Sarda and Dhanak, 2018). These mobile solutions depend on a similar design to pole docking solutions. To facilitate docking, the AUV has a pair of pincers mounted at the nose of the vehicle which capture a submerged taut cable. While these designs work well for recovery, they do not allow easy integration of power transfer electronics, require external actuation of the pincer mechanism, and need to be mounted at the front of an AUV which is critical for scientific and navigational sensors.

Additional inspiration for undersea docking design and control can be gathered from assessing docking technology in other domains and missions. Specifically, underwater intervention, surface docking, and docking between ground vehicles all operate on the same fundamental principle of controlling vehicle rendezvous. In underwater intervention missions, an overactuated hovering type AUV is deployed to interact with an undersea panel (Palomeras et al., 2014) or docking station (Bianchi Figueiredo and Coimbra Matos, 2020). The hovering AUV then grapples to the panel and docks (Palomeras et al., 2014). This type of application, while initially appearing similar to the proposed mission is not directly transferable to the target application due to the underactuated nature of traditional AUVs and lack of articulated arms. Surface and ground vehicle docking is more applicable to the proposed mission as underwater operation can be considered a two dimensional problem if operating at a fixed depth. On the surface, many docking techniques exist from the fully actuated (Mateos et al., 2019) to the underactuated (Xie et al., 2018). Ground has seen significantly more research over the decades with solutions ranging from mechanical (Silverman et al., 2002) to non-contact (Lu et al., 2018). One common theme across surface and ground applications is the higher quality of localization information and relatively higher control authority available to the platform.

**FIGURE 1:**
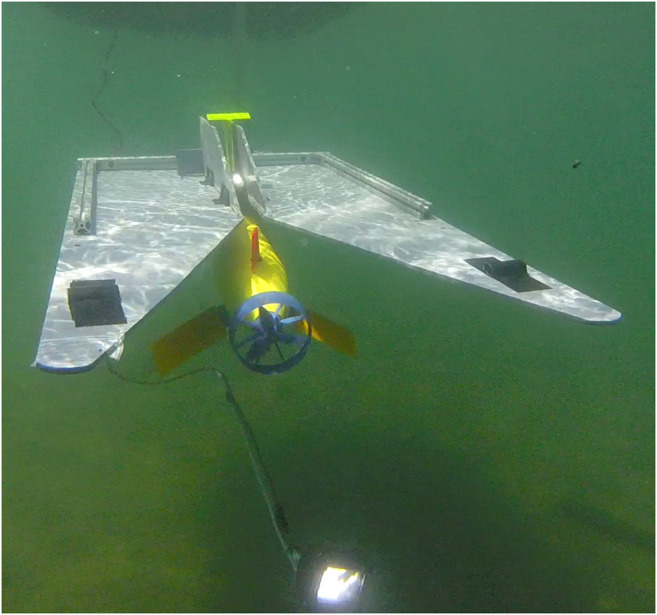
The flat funnel docking system uses a single beacon-single camera approach to guide the AUV into the docked position. The docking system is adaptable to a variety of AUVs and collapsible for easy transport.

In this paper, the docking system detailed in (Page and Mahmoudian, 2020) was used. The docking system consists of two components, the docking station and a docking adapter mounted to the AUV. The docking station is capable of supporting a wide range of AUVs through its novel use of a simplified funnel design and docking adapter. This design idea simplifies the traditional fully three dimensional funnel design into two pieces, a flat funnel and a ramp. Further, the use of the docking adapter enables AUVs of different size classes to dock with one common station. Figure 1 shows the dock, beacon light, and AUV. With the simplified docking design, we are able to use a simple controller to achieve relatively good performance as we decouple control in the vertical and horizontal axis. A detailed discussion of the docking system is available in (Page and Mahmoudian, 2020) including modeling and design optimization.

In general, the docking process is broken into five stages (Bellingham, 2016):

1. En route: the vehicle approaches the dock using long range navigation such as dead reckoning.
2. Approach setup: once within range of the near navigation sensors such as USBL the vehicle navigates to the start of the docking trajectory.
3. Approach: the vehicle follows the approach trajectory to the docking station.
4. Terminal homing: when near the docking station, the vehicle attempts to further improve its docking accuracy through the use of high update rate sensors such as vision.
5. Capture: the vehicle impacts the docking station and maintains thrust until latched.

The en route stage is the typical operating mode for an AUV where it will navigate towards waypoints using standard navigational sensors such as GPS, compass, and Doppler Velocity Log(DVL). Due to the internally estimated nature of most marine navigation, occasional surfacing is required to eliminate unbounded localization error during submerged operation.

Depending on the application, surfacing might not be desirable, especially for long term missions when the cost in terms of time and energy is high (Dhanak and Xiros, 2016). In this scenario, acoustic ranging methods have proven effective (Morgado et al., 2014; Caiti et al., 2014; Nad et al., 2016) in keeping the error bounded while continuing the mission submerged.

Acoustic ranging methods involve the use of at least two beacons (or transponders) to get an absolute position through acoustic communication. In this field, two types of systems have been primarily explored: Long Baseline (LBL) and Ultra-Short Baseline (USBL) (Dhanak and Xiros, 2016). The first requires two or more beacons deployed in fixed locations prior to the AUV deployment. In comparison, the USBL method only requires two beacons since one of them contains an array of transducers that estimate position based on a phase difference of the received signal. These acoustic methods are also critical to enable an accurate approach setup and approach phase for docking, especially when considering docking in the deep ocean or while tracking a moving dock.

The docking maneuver has seen different control approaches over recent years, with the majority of work focusing on terminal homing. The simplest docking algorithms are based on pure pursuit where the vehicle aims directly at the docking station (Bellingham, 2016; Yazdani et al., 2020). Some control algorithms have attempted to compensate for the effects of currents. A combined pursuit-proportional navigation algorithm (Yang et al., 2016), hybrid sliding approach (Sans-Muntadas et al., 2016), fuzzy control (Teo et al., 2012), and cross-track based control (McEwen et al., 2008) have proved their effectiveness at docking in currents. More recent docking work has been completed across a range of problems including high-rate localization (Cheng et al., 2020), terminal homing (Zhang et al., 2019), physical docking (Matsuda et al., 2019; Yan et al., 2019), and simulation (Meng et al., 2019; Wu et al., 2019).

Cross-track error based controllers have the benefit of being computationally simple to implement while maintaining adequate performance. During the docking maneuver, localization of the docking station can be done visually (Li et al., 2016), magnetically (Feezor et al., 2001), or acoustically (McEwen et al., 2008). Each of these localization methods has unique benefits with visual based methods generally having the highest accuracy and update rate in close proximity of the dock in good visibility. No docking control strategy is able to ensure a 100% docking success rate, so the AUV must be able to detect failures and go for re-attempt (Page and Mahmoudian, 2020). Failures in open water typically are caused by currents and incorrect localization information.

**FIGURE 2:**
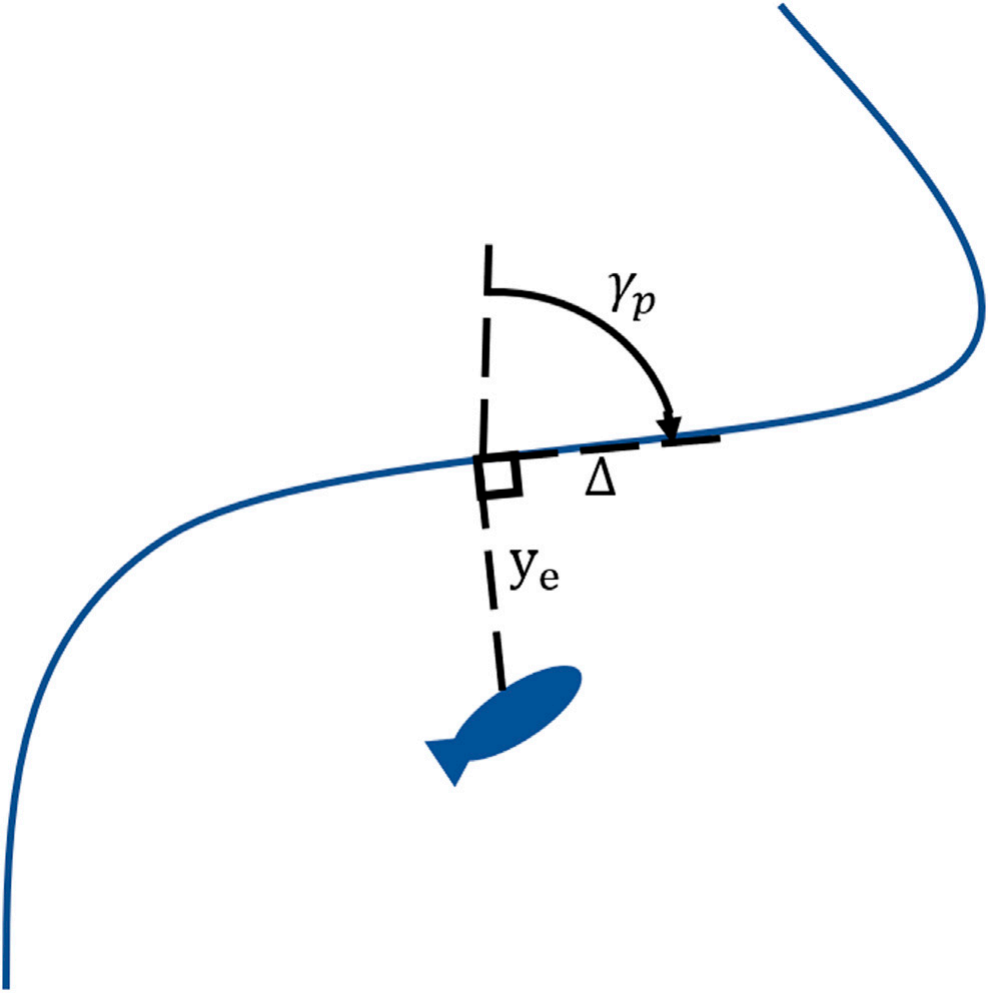
Line of sight controller parameters. $y_{e}$ is cross track error, Δ is lookahead distance, $\gamma_{p}$ is projected heading.

When considering a larger scale to include the docking approach setup and approach phases, the path followed by an AUV can generally be thought of as a 2D trajectory with a fixed depth. Further, AUVs can traditionally be considered as Dubins cars capable of traveling in straight lines and turns up to a maximum rate (Scibilia et al., 2012). These two assumptions on vehicle operations enables the applications of Dubins set to the vehicle planning process (Dubins, 1957). The solution of the Dubins set is well documented and efficiently calculated and results in a smooth trajectory from an initial position and orientation towards a final position and orientation (Shkel and Lumelsky, 2001). The trajectory will always consist of a series of either Turn-Straight-Turn or Turn-Turn-Turn. The trajectory can then be followed using an integral line of sight (ILOS) control strategy (Fossen et al., 2015; Caharija et al., 2016). In this strategy, the AUV calculates a desired heading based on its current pose and the target path. The desired heading is fed into the vehicle frontseat controller for reference tracking.

$$\phi_{D}=\gamma_{P}+\tan^{-1}\left(-\frac{1}{\Delta }y_{e}-\hat{\beta }\right),$$ (1)

where $\phi_{D}$ is the desired heading, $\gamma_{P}$ is projected heading, Δ is the lookahead distance, $y_{e}$ is cross track error (Figure 2), and $\hat{\beta }$ is the estimate of sideslip angle based on the integral of Eq. 2. Lookahead distance (Δ) is user selected and can be based on an adaptive controller where the input is $\alpha =\Delta \hat{\beta }$ subject to $0<\Delta_{\min}\leq \Delta \leq \Delta_{\max}$.

$$\dot{\hat{\beta }}=\gamma \frac{U\Delta }{\sqrt{\Delta^{2}+{\left(y_{e}+\Delta \hat{\beta }\right)}^{2}}}y_{e},\gamma >0,$$ (2)

where *U* is vehicle forward velocity and γ is adaptation gain.

## 3 Algorithm Design

The docking algorithm is implemented in two stages. The first stage is the approach navigation capable of supporting dynamic replanning. The second stage is terminal homing. These two methods combine to create an integrated docking algorithm capable of supporting docking operations in challenging environments. Implementation of the algorithm on the backseat of any frontseat-backseat controlled vehicle allows use irrespective of vehicle dynamics and frontseat control methodology.

### 3.1 Approach Navigation and Replanning

Navigation while operating on the approach setup and approach phases of docking is critical for the overall success of any docking procedure. In this work, Dubins paths (Dubins, 1957; Shkel and Lumelsky, 2001) and integral line of sight navigation (ILOS) (Borhaug et al., 2008; Fossen et al., 2015) are combined to create an efficient path planning/following method capable of online re-planning to account for intermittent localization updates and failed docking attempts. The output of this proposed method is a simple reference heading that can be tracked by an existing navigational controller onboard the vehicle.

The proposed method operates in three stages: path creation, path augmentation, and path following. In the path creation stage, the Dubins set is leveraged to create a smooth trajectory between target waypoints. This prescribed path is the desired path that the AUV should follow for the duration of the mission. The path creation stage runs at mission initialization and whenever the docking procedure is triggered. Path augmentation involves the vehicle position being projected onto the current path. If the cross track error from the current path is unacceptably large then a new Dubins rendezvous trajectory is drawn from the current location towards a point on the prescribed path at a constant lookahead distance. The current path is then updated with the new rendezvous trajectory. In the path following stage, the current path is followed using a standard integral line of sight control strategy. The algorithm follows the steps prescribed in Algorithm 1.

**Table:** 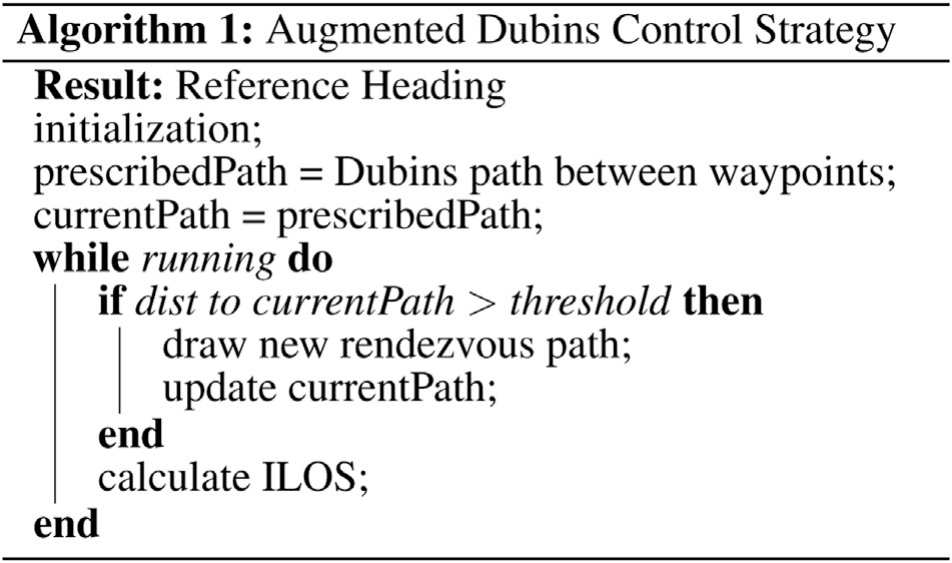

Effectively, the control strategy simplifies down to using integral line of sight control to follow a Dubins path. However, if the Dubins path is far from the current position, such as can happen when surfacing or after a failed docking attempt, a new rendezvous path will be generated. The use of rendezvous Dubins paths guarantees that the integral line of sight controller never needs to drive the vehicle to converge to the prescribed path from far away and enables the path following controller to be tuned to aggressively track the path with much less strict bounds on stability.

The proposed algorithm is capable of driving the vehicle during normal operation as well as during docking approach. When in normal operating mode, the set of waypoints is prescribed by a mission planner such as (Li et al., 2019). When in docking mode, the set of waypoints is calculated based on projecting a straight line through the known docking location. This enables a smooth handoff to the terminal homing process. Note: the docking location can be known a-priori as with fixed docking or can be transmitted using acoustic means to allow the vehicle to approach towards a moving target.

### 3.2 Terminal Homing Control

**FIGURE 3:**
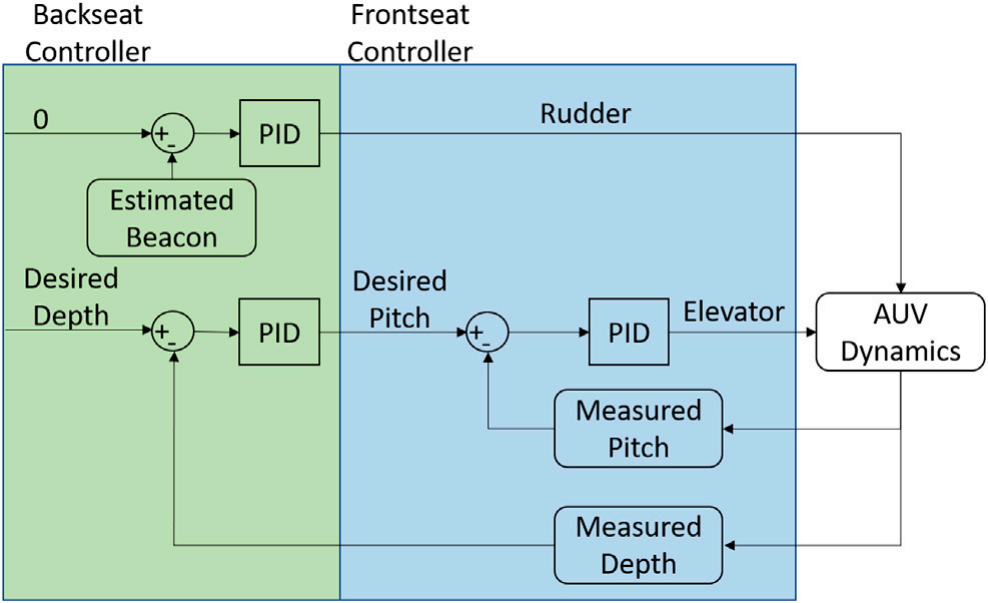
Frontseat-backseat control architecture used on the AUV to complete docking. The frontseat controller is the navigation controller onboard the vehicle while the backseat controller is located in the payload.

**FIGURE 4:**
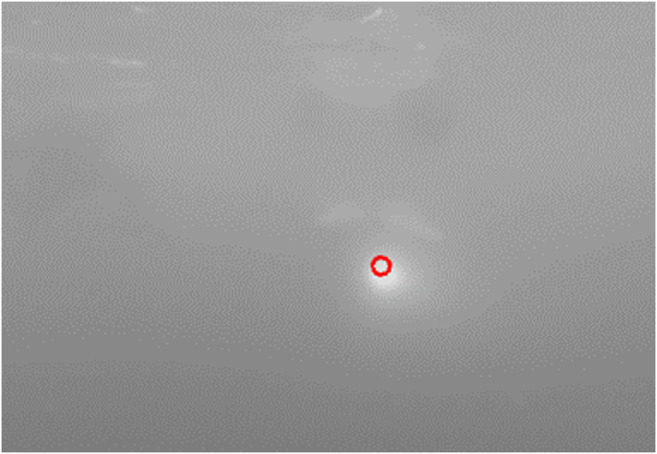
Output of the image processing algorithm. The horizontal distance in pixels from the center of the frame to the target is fed into the controller.

Backseat control in the terminal homing stage is broken into the dive plane and the longitudinal plane, Figure 3. In the dive plane, the AUV attempts to achieve an a-priori known docking depth using a nested pitch-depth Proportional-Integral-Derivative (PID) controller. In the outer loop, the backseat controller calculates a target pitch angle based on the reference depth and measured depth using a pressure sensor. The target pitch is then fed into the frontseat controller which uses an independent PID controller to drive the vehicle towards the desired pitch. In the longitudinal plane, the backseat controller uses a single webcam to look for a single beacon. Image processing is completed as a three step process, Figure 4:

1. Grab gray scale image
2. Gaussian blur
3. Pick brightest pixel

The three step image processing process is efficient enough to run on many single board computers without excessive computational load while displaying sufficient performance in initial testing.

This simple image processing algorithm can suffer from false positives if operating in a bright environment such as near the surface during the day. This has occurred during experimental testing, however, while operating in deep water or at night, the beacon light is the dominant source of illumination. Further work is required to remedy the surface interaction issue. More advanced processing algorithms have been attempted including a k-means tracker (Kanungo et al., 2002), Hough circularity transform (Yuen et al., 1990), and the SimpleBlobTracker in OpenCV (Bradski and Kaehler, 2000). While these processing algorithms have more customizability for specific scenarios they have experimentally not shown to be as robust to changes in water clarity as the described three step processing method utilized in this work.

Once the location of the beacon has been calculated, the offset from the center of the camera frame is fed into a PID controller that attempts to drive the beacon towards the center of the image by changing vehicle yaw (rudder). Overall, this control strategy is as simplified as possible to limit the experimental tuning required and decrease computational load. If at any point the terminal homing method is unable to acquire the target, the docking attempt is aborted and the re-plan method is triggered to drive the vehicle towards a re-attempt.

## 4 Experimental Setup and Results

Validation of the proposed docking algorithm was completed in two phases. In the first phase, an OceanServer Iver3 was utilized to verify the ability of the approach navigation algorithm to maneuver an AUV to a terminal homing handoff location. In phase two, a Bluefin SandShark was used to validate the terminal homing algorithm’s ability to dock from such a handoff location. The SandShark was chosen due to its small scale which enabled testing and experimental deployment in a pool environment. For the purposes of this work, we assume that the vehicle has some sort of onboard localization estimate such as through dead-reckoning or acoustics. Additionally, a forward facing camera is assumed to be available along with sufficient processing overhead to run a rudimentary vision algorithm.

### 4.1 Approach Navigation

Navigation from the far field towards a docking location occurs broadly in two stages: approach and terminal homing. Success of the approach phase is critical for overall docking performance. With a goal of delivering the vehicle towards a control handoff location that is directly in front of the known docking location.

#### 4.1.1 Experimental Setup

Experimental validation of the approach navigation algorithm utilized the OceanServer Iver3. The Iver3 was chosen due to its proven mission robustness and use by operators in the field. The vehicle was deployed near Purdue University’s campus at Fairfield Lakes Park in a body of water approximately 700 m by 170 m at its widest.

The Iver3 is approximately 2 m long and 30 kg and is capable of operating at speeds up to four knots for approximately 12 h. For navigation, the vehicle features two identical compute modules. The primary (frontseat) controller comes from the manufacturer with basic navigation software that fuses sensor information and is capable of driving the vehicle towards waypoints for lawnmower navigation. The secondary (backseat) controller is user configurable and interfaces with the frontseat controller using NMEA messaging (Brown, 2018). This frontseat-backseat configuration enables the vehicle to operate in frontseat navigation mode during the majority of the mission, with the backseat taking over during complex maneuvers.

Approach algorithm validation with the Iver3 used a user defined “virtual” coordinate to represent a terminal homing controller handoff location to allow controlled development to focus on improving the approach phase independently. Additional preliminary testing was conducted using acoustic communication to update the docking handoff location during runtime to track a slowly moving docking location.

#### 4.1.2 Approach Validation

**FIGURE 5:**
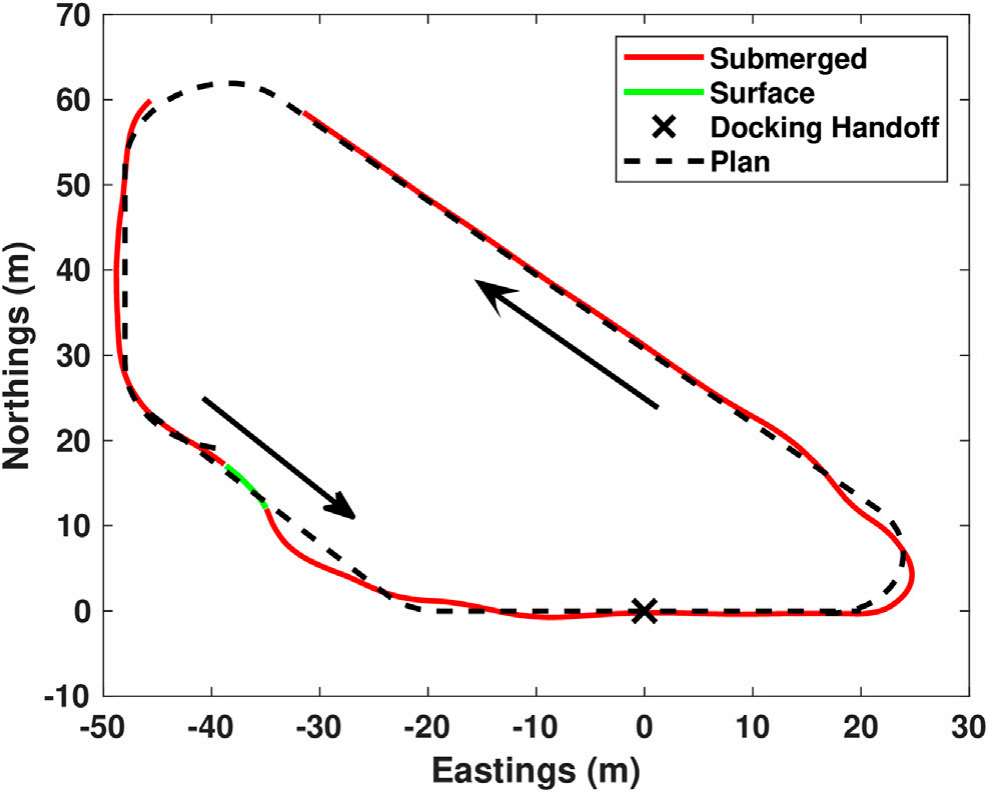
Virtual docking handoff testing. In this plot, red is submerged, green is surfaced operation, black is the plan. Presented here is a single lap of the test trajectory. There are three plans presented here. The first plan consists of the majority of the operation around the loop and ends at the point (-40,20,0) (x,y,orientation). Upon reaching the end of the planned trajectory, the system re-plans a Dubins path from there to the approach vector which is a straight line from −20 to +20.

**FIGURE 6:**
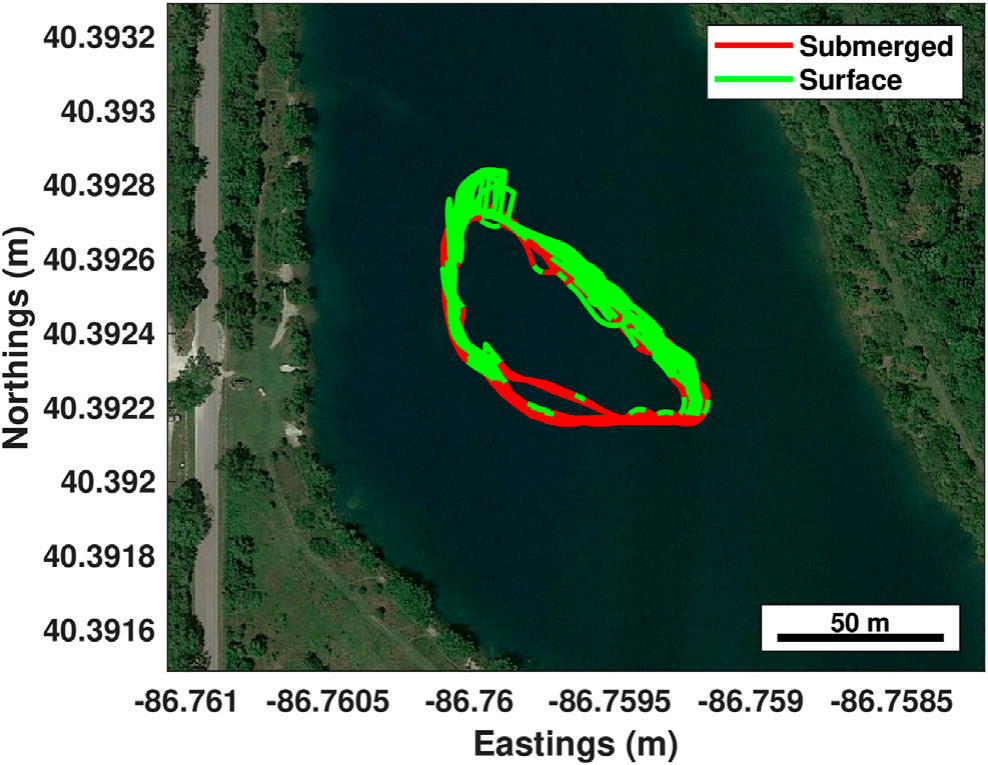
Virtual docking handoff testing over 3 h. A short trajectory was chosen to focus more on the docking process and replan-ability of the algorithm vs long range navigation capabilities. In this plot, red is submerged, green is surfaced operation. The jumps in estimated localization are due to the GPS re-localizing on surfacing.

**FIGURE 7:**
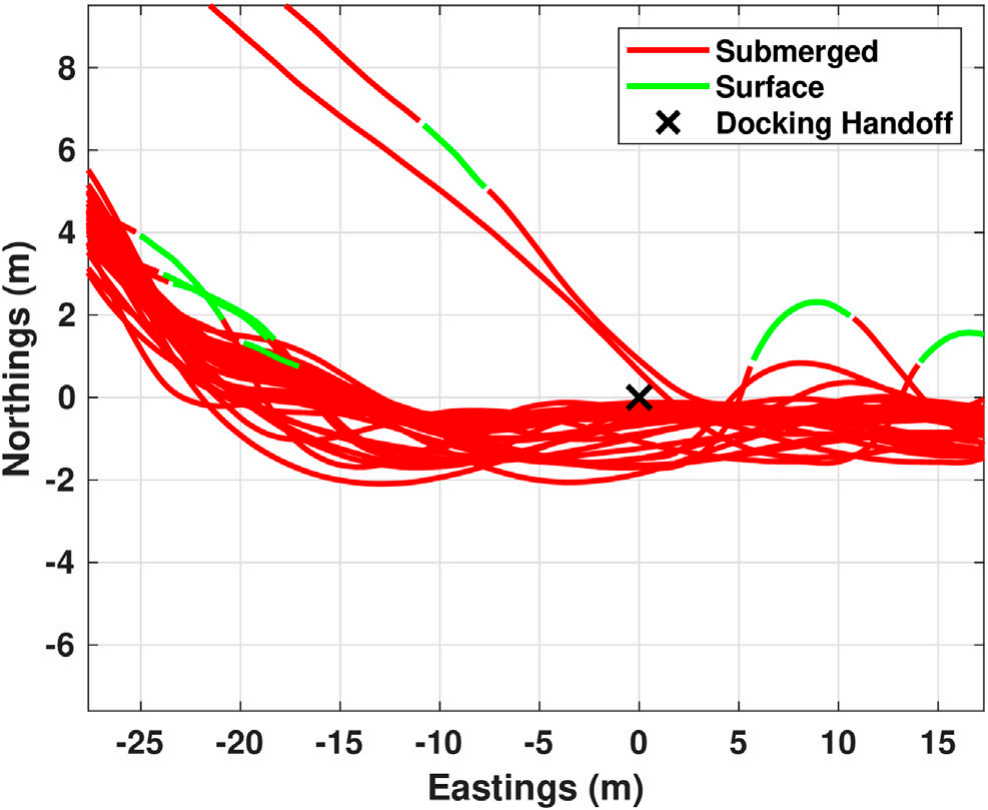
At the virtual docking handoff, 39 out of 48 tests were within 1 m, and all 48 were within 2 m. This will result in high success rate once terminal homing is added.

The vehicle was deployed on a short distance trajectory with one “virtual” docking handoff attempt approximately every 3–4 min to increase the number of trials rapidly. During testing, the algorithm drove the vehicle along the planned trajectory with 120 s submerged periods followed by 45 s on the surface. Once the docking trajectory was triggered, the vehicle drove on the surface until 20 m from the docking control handoff location, it then continued on a submerged linear path through the handoff location that would be where a terminal homing controller would drive the vehicle into a hard dock. Figures 5–7 demonstrate validation of the approach navigation algorithm through a virtual docking scenario.

Implementation of the algorithm on the backseat controller was completed using *Python* with a custom Robot Operating System (ROS) package that interfaced over the Iver3 communication link. The frontseat controller was polled at regular intervals for vehicle state information (position, orientation, etc). This state information was fed into the algorithm. Received positions were converted to UTM coordinates and compass bearings were converted into geometric angle from +X. For the small scale testing, the prescribed path was a short Dubins path North of the docking location. The docking path consisted of a linear alignment vector of 20 m at an angle of 0° from +X going through the (0,0) point. In the general case, this would be a line projected from the docking heading.

Data presented in this section is the onboard vehicle estimate. Since the Iver3 has the addition of a doppler velocity log, accurate compass, and better vehicle model. The vehicle estimates are considered to be accurate enough for the purposes of this work due to the frequently commanded GPS surfacings and due to the terminal homing process’ ability to reject any minor errors. The addition of external localization estimation such as USBL will eliminate the need for re-surfacings as well as allow the docking handoff location to be updated during operation to track a moving dock. To accelerate development of the re-plan method, the Iver3 compass calibration was not completed in the Fairfield Lakes pond. This resulted in a slight heading error in the tests and caused more re-plans to be triggered during the test.

In the presented 3 h test, 48 virtual docking attempts were completed. The planner updated the path 137 times. The re-plan was triggered primarily on re-localizing after surfacing as well as at the start of, and end of each docking attempt. With the re-plan method, the vehicle was able to navigate to within 1 m of the prescribed virtual handoff point in 39 of the 48 attempts, and within 2 m in all 48 attempts. This very high degree of success indicates that the approach navigation algorithm is a good candidate for a unified docking control strategy. When combined with the later presented terminal homing results, we expect an open water hard docking success of greater than 70%. With re-attempts included, this success rate rapidly improves assuming the scenario is feasible.

Verification of the algorithms ability to adjust to a dynamically defined handoff location was also tested. This testing included an Autonomous Surface Vehicle (ASV) equipped with a SeaTrac X150 USBL and a docking station. A SeaTrac x110 acoustic modem was integrated with the Iver3. This provided the vehicle with communication (100 bps) and ASV relative positioning capabilities (accuracy within 1.5% of beacon separation distance) (Neasham et al., 2015).

The updated position and heading information of the ASV was sent periodically (4s) to the Iver3, which received the position through the SeaTrac X110 acoustic modem. The new position and heading information was fed into the navigation algorithm to calculate a path to align the AUV with the docking station. Preliminary testing showed that the algorithm has the capability to adapt to a mobile docking position. However, further testing and evaluation with active handoff to a terminal homing procedure is required for full validation.

### 4.2 Terminal Homing

**FIGURE 8:**
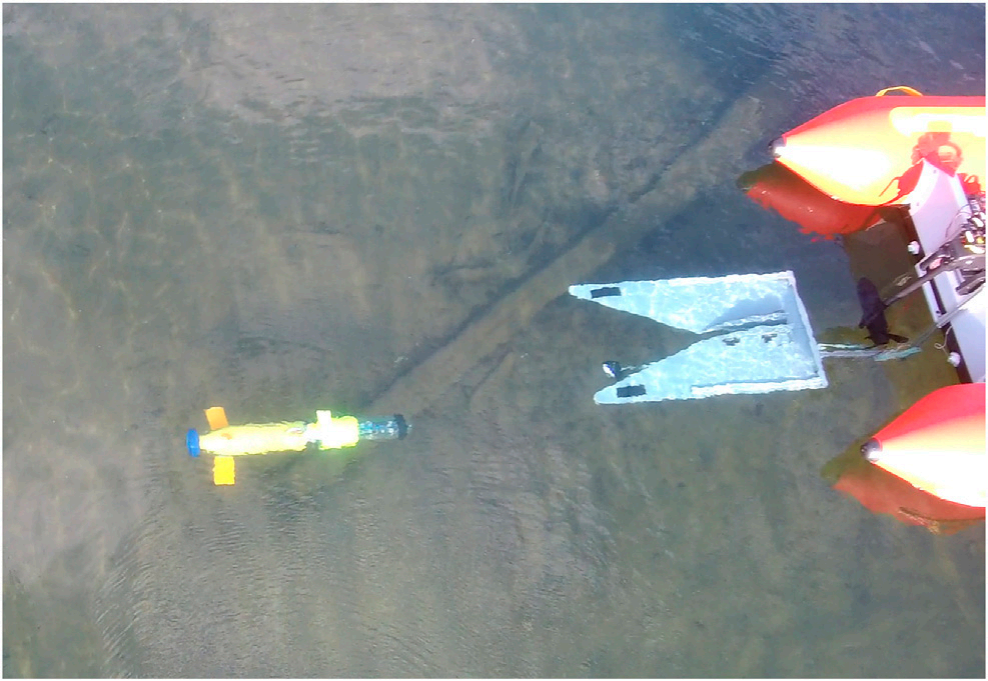
AUV approaching docking station during a mission.

The terminal homing algorithm has been tested with the adaptive docking system both indoors and in open water in/near the facilities of Michigan Technological University. Presented is the experimental setup and data analysis strategy for homing validation as well as the validation results. Validation was done in three subsequent stages: 1) indoor fixed docking, 2) indoor mobile docking, and 3) outdoor (open-water) docking using a Bluefin SandShark, Figure 8. The SandShark was used due to its small scale which makes pool development feasible.

#### 4.2.1 Experimental Setup

Development and testing of the terminal homing stage was completed in two phases indoors. In the first phase, the docking station and associated beacon light was rigidly attached to the pool wall. The AUV was then released from the opposite pool wall at varying initial positions and headings. For mobile docking, the docking station and beacon light were attached to the rear of a small inflatable dinghy. The boat was then manually rowed in approximately linear paths across the pool while the AUV attempted docking. The highly variable nature of manual rowing is roughly similar to the disturbances experienced in open water. Open water docking was completed with the dock mounted and beacon light at the rear of the inflatable. In the open water case, the inflatable was performing manual station keeping. Indoor docking trajectories were recorded using an overhead camera setup and outdoor trajectories are vehicle estimates. This subsection describes the processing completed to calculate indoor trajectories.

The overhead camera setup utilized a fixed overhead GoPro camera. The camera recorded the entire pool scene during all indoor testing. Videos from the tests were post-processed in MATLAB using a four step process to calculate pool trajectories:

1. Undistort Fisheye
2. Rotate and Project
3. Crop to Pool Region
4. Identify Targets using Color

In the first step, individual frames are undistorted according to (Scaramuzza et al., 2006) using the MATLAB *undistortFisheyeImage* function. This tool removes the lens distortion, however, the shape of the pool in the resulting image is still trapezoidal due to the camera not being mounted directly above the center of the pool. A projective transformation was then applied to the image to rectify the image (Hartley, 1999) prior to cropping. The rectified pool image was then processed to identify targets based on color. For the fixed docking trials this meant identifying just the AUV location as the docking station was manually set. For the mobile trials, this meant identification of both the AUV and mobile dock. To locate the AUV, the rectified image was first converted into YCbCr color space. The converted image was then masked to focus on the yellow component of the AUV. Locating the mobile dock proved to be much more challenging than the AUV as it has a much more muted color. The current best method is to Gaussian blur the rectified image with a standard deviation of 5, then mask in RGB space to identify the dark green color of the boat. The masked images were then processed using the *regionprops* tool to record the location of the largest masked region. Frames were processed sequentially and output both a processed video with overlaid targets and numerical trajectory logs.

#### 4.2.2 Docking Validation

**FIGURE 9:**
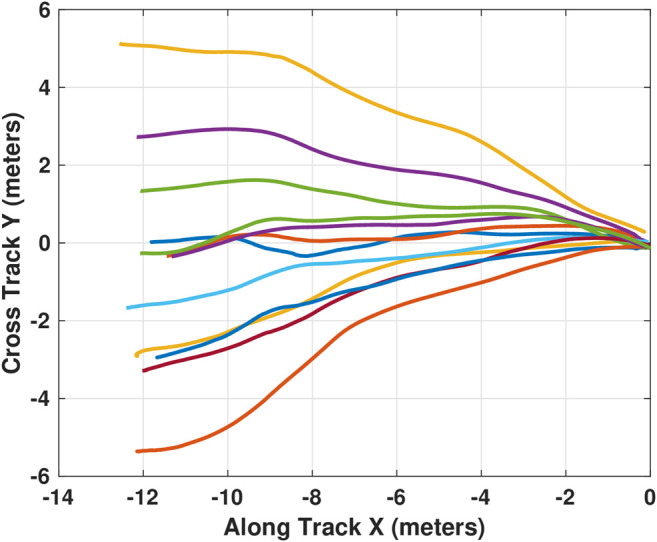
Trajectories followed during testing of docking to a fixed target. 12 successful docking attempts are shown here converging to the dock located at (0,0).

Initial experimental control development and evaluation was completed to a fixed docking station mounted on the side of the Michigan Tech dive pool. This docking scenario is equivalent to a seafloor mounted docking station. Evaluation of docking performance was completed by releasing the AUV from a range of initial locations and headings. Figure 9 shows the trajectories followed during testing. In the figure, the AUV was released over the full width of the pool (11.88 m) and approximately 12 m from the dock. The AUV was able to dock with a success rate of approximately 70% (12 of 17 trials). Failed docking attempts occurred in the pool 30% of the time as the localized jets were quite strong and could sometimes disturb the vehicle during the final few meters of the trajectory. These localized currents are not encountered in open water.

From Figure 9, we can see that the pure pursuit based controller is capable of successfully docking when there is little disturbance. However, it is also clear that the controller will attempt to drive directly into the dock from wherever it is released. This direct approach works well when released within a relatively narrow conical projection from the ideal approach vector but may cause problems if released with a relatively large cross track error. This result indicates the need for an accurate approach phase.

**FIGURE 10:**
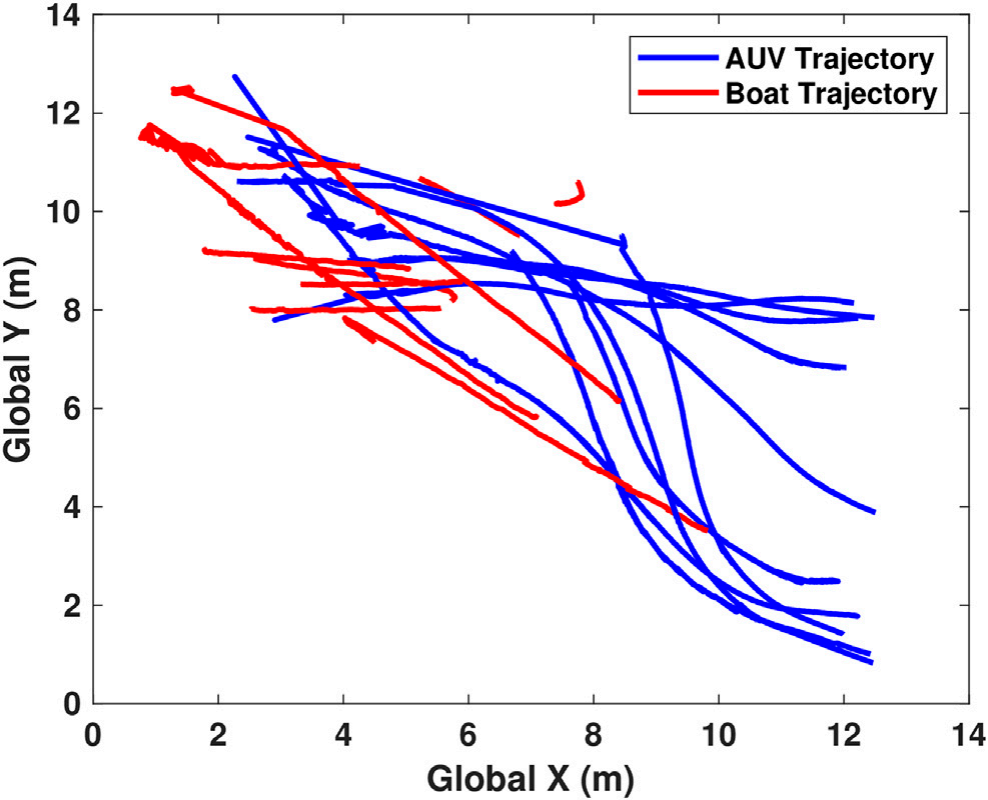
All trajectories of both the AUV and boat during mobile testing. Red is boat, blue is AUV.

**FIGURE 11:**
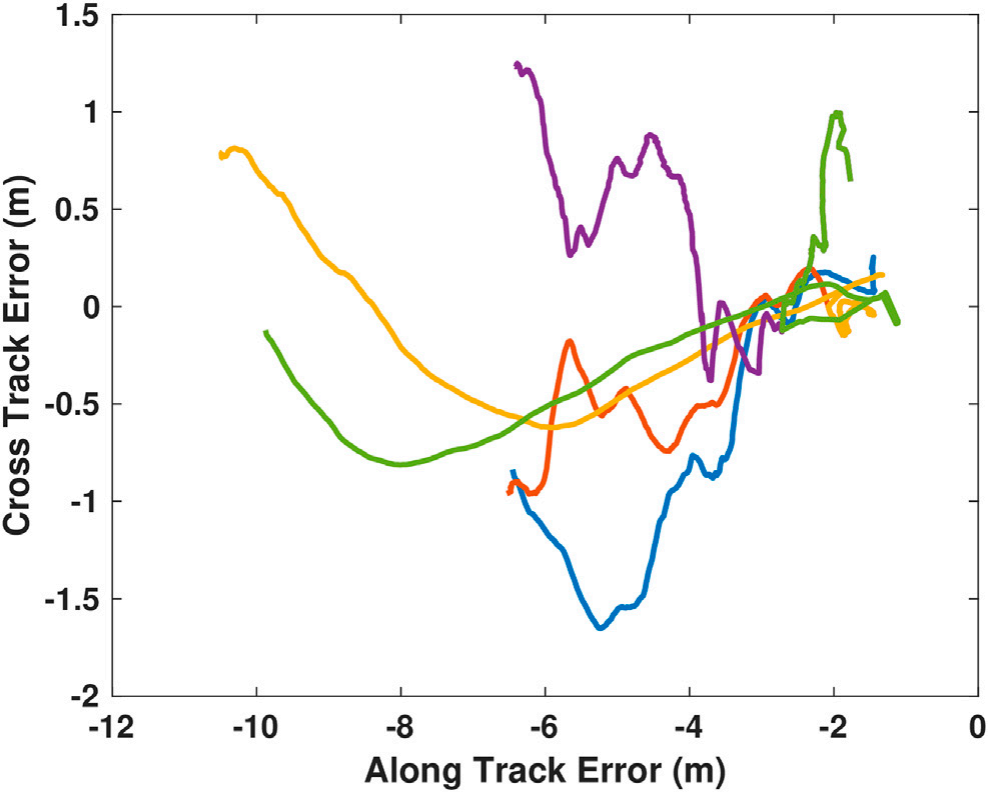
All successful local trajectories followed by the AUV during mobile docking. The trajectories are more varied due to the combined action of both the AUV and the rowed boat.

Following the success of the docking procedure to a fixed docking station, a more challenging scenario was attempted. In this scenario, the AUV was released from one side of the dive tank while the docking station was mounted to a manned dinghy. The dinghy was manually rowed in approximately linear paths towards and away from the AUV to evaluate controller performance. Figure 10 shows all the trajectories overlaid. To ease visualization of the actual docking trajectory, the motion of the AUV is also presented in terms of the docks local coordinate system, Figure 11. In the presented scenario, the AUV was able to successfully dock to the moving target with a 50% success rate (5 of 10 attempts).

In the local, successful trajectories (Figure 11), the approach trajectories are much more erratic compared to the successful static trajectories (Figure 9). This is due to small rotations (yaw) of the mobile docking station during approach.

**FIGURE 12:**
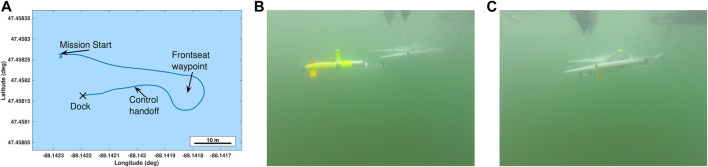
**(A)** Onboard vehicle estimate during one of the open water docking approaches. **(B)** The AUV transits 40 m away from the dock, turns around, then approaches the docking station. **(C)** It then impacts the station with the docking adapter and slides towards the docked position.

**FIGURE 13:**
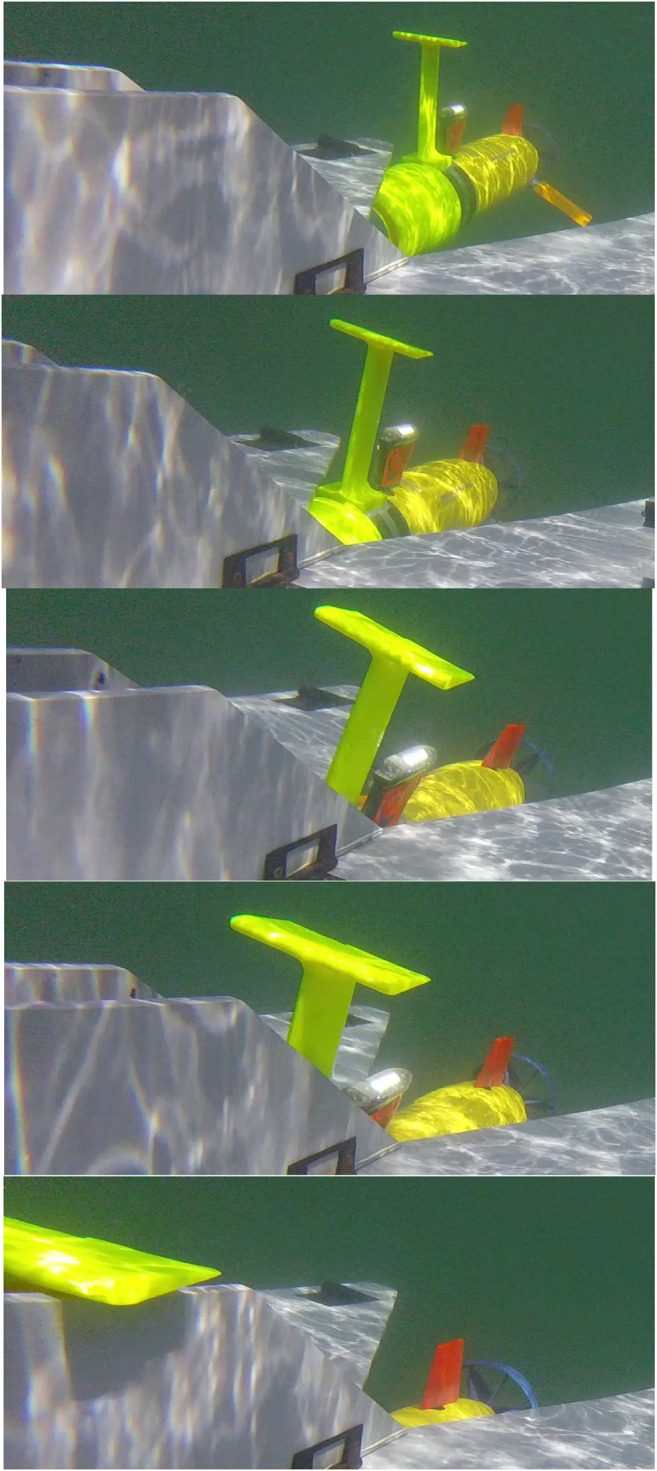
The docking adapter mounted on the AUV interacts with the docking station during final approach in open-water.

Initial open water testing was completed in Lake Superior to evaluate the transition of the developed dock and terminal homing technique to real world conditions. Figure 12 shows the docking process in open water. In Figure 12A, the AUV was released near the surface vessel to which the docking station was mounted. It then transited towards a waypoint under frontseat control away from the dock before turning around to align with a control handoff waypoint. On the terminal approach, the frontseat controller signaled the handoff to the backseat controller. The backseat then calculated a pure pursuit based terminal homing trajectory. During all open water testing, the dock was mounted onto a small (2 m) manned dinghy performing manual station keeping. A sample of docking is shown in Figure 13, please see for video of multiple successful outdoor docking attempts. The overall successful docking rate was significantly lower in open water. Challenges that arose during the transition to open water included visibility limitations, motions due to waves and currents, and inaccurate docking handoff locations. With the reduced visibility in open water we were able to acquire the target when approximately 9 m away at maximum. This was limited during algal blooms and also resulted in false positive events when the Sun was identified as the dock. The primary limiting factor in the direct transition of the terminal homing algorithm towards open water was limited accuracy in the frontseat—backseat handoff locations for docking.

The terminal homing validation focused exclusively on the terminal homing process using a single-camera, single-beacon approach. The results of this testing validated that the approach navigation algorithm is necessary for real-world docking. Integration of acoustic methods and approach navigation as prescribed in this work are required to achieve satisfactory real-world terminal-homing performance.

## 5 Conclusion

Collaborative fleets of AUVs that have the ability to reliably and repeatedly complete autonomous underwater docking can operate without interruptions on current and future undersea missions. This paper presented a docking navigation algorithm consisting of approach and terminal homing algorithms. The approach algorithm is capable of dynamic re-planning to account for disturbances and failed docking attempts while accurately delivering the vehicle to a terminal homing control handoff location. In the tested scenario, 48 of 48 tests delivered the AUV to a suitable handoff position and orientation to enable docking. The terminal homing algorithm is an intentionally simplified visual approach capable of successfully docking to the adaptive docking station in 70% of test scenarios assuming acceptable initial conditions from the approach. When combined, these two methods will enable long term persistence of autonomous underwater vehicles through efficient navigation and accurate docking operations. In the event of a missed docking attempt, the algorithm has the inherent ability to go around and re-attempt. This will quickly drive the overall success rate asymptotically towards 100%, with a 99% chance of successful docking by the fourth attempt assuming the operating conditions are similar to the tested environment.

Experimental validation of this navigation algorithm was implemented onto a Bluefin SandShark and Oceanserver Iver3. The SandShark was used for terminal homing due to its small scale which enabled pool based tuning and debugging of the terminal homing method. The Iver3 was used for approach navigation due to its long real-world operational history.

Going forward, these methods are being fully integrated onto the Oceanserver Iver3 to create an end-to-end navigation solution capable of navigation on complex mission scenarios including multiple working AUVs, continuous submerged operation, and charging surface vehicles. The integrated navigation solution will be able to drive the vehicle through the entirety of the mission and support persistent operation with fixed underwater or mobile surface recharging systems. This will enable true resident AUV operation.

Future work on this project is extensive. Specifically, the proposed terminal homing localization method is meant as a first approximation for a docking system to validate system performance. Following extensive testing and development, visual data will be collected using external acoustic localization as ground truth. A deep learning approach will then be implemented to learn to localize to the dock in six degree of freedom space based on the vision system. Further, the approach method proposed here operates at a fixed depth. The addition of variable depth control and planning will help extend the method to more general mission planning and operation. We will also explore optimizing the algorithm further with adaptive choice of handoff location, re-plan thresholds, and lookahead distance.

## Data Availability

The raw data supporting the conclusion of this article will be made available by the authors, without undue reservation.

## References

[B1] Allen B. Austin T. Forrester N. Goldsborough R. Kukulya A. Packard G. (2006). Autonomous docking demonstrations with enhanced remus technology. IEEE. In OCEANS 2006, Boston, MA, United States, 18-21 September 2006. 1–6. 10.1109/OCEANS.2006.306952

[B2] Bellingham J. G. (2016). Autonomous underwater vehicle docking. Cham, Switzerland: Springer International Publishing, 387–406. 10.1007/978-3-319-16649-0_16

[B3] Bianchi Figueiredo A. Coimbra Matos A. (2020). Mvido: a high performance monocular vision-based system for docking a hovering auv. Appl. Sci. 10, 2991. 10.3390/app10092991

[B4] Borhaug E. Pavlov A. Pettersen K. Y. (2008). Integral los control for path following of underactuated marine surface vessels in the presence of constant ocean currents. In 2008 47th IEEE Conference on Decision and Control, Cancun, Mexico, 9-11 December 2008. 4984–4991. 10.1109/CDC.2008.4739352

[B5] Bradley A. M. Feezor M. D. Singh H. Yates Sorrell F. (2001). Power systems for autonomous underwater vehicles. IEEE J. Ocean. Eng. 26, 526–538. 10.1109/48.972089

[B6] Bradski G. Kaehler A. (2000). Learning OpenCV 3: computer vision in C++ with the OpenCV. Sebastopol, CA: O’Reilly Media.

[B7] Brighenti A. Zugno L. Mattiuzzo F. Sperandio A. (1998). Eurodocker-a universal docking-downloading recharging system for auvs: conceptual design results. In IEEE Oceanic Engineering Society. OCEANS’98. Conference Proceedings (Cat. No.98CH36259), Nice, France, 28 September-1 October 1998. 3, 1463–1467. 10.1109/OCEANS.1998.726313

[B8] Brown H. C. (2018). Iver3 benchseat driver for virtual remote helm functionality. OCEANS 2018 MTS/IEEE Charleston, Charleston, SC, United States, 22-25 October 2018. 1–6. 10.1109/OCEANS.2018.8604715

[B9] Caharija W. Pettersen K. Y. Bibuli M. Calado P. Zereik E. Braga J. (2016). Integral line-of-sight guidance and control of underactuated marine vehicles: theory, simulations, and experiments. IEEE Trans. Contr. Syst. Technol. 24, 1623–1642. 10.1109/tcst.2015.2504838

[B10] Caiti A. Di Corato F. Fenucci D. Allotta B. Costanzi R. Monni N. (2014). Experimental results with a mixed usbl/lbl system for auv navigation. IEEE, In Underwater Communications and Networking (UComms) 2014, Sestri Levante (Genova), Italy, September 2014, 1–4. 10.1109/UComms.2014.7017129

[B11] Cheng H. Chu J. Zhang R. Gui X. Tian L. (2020). Real-time position and attitude estimation for homing and docking of an autonomous underwater vehicle based on bionic polarized optical guidance. J. Ocean Univ. China 19, 1042–1050. 10.1007/s11802-020-4399-z

[B12] Dhanak M. R. Xiros N. I. (2016). Springer handbook of ocean engineering. Berlin, Germany: Springer. 10.1007/978-3-319-16649-0

[B13] Dubins L. E. (1957). On curves of minimal length with a constraint on average curvature, and with prescribed initial and terminal positions and tangents. Am. J. Math., 79 , 497–516 10.2307/2372560

[B14] Feezor M. D. Yates Sorrell F. Blankinship P. R. Bellingham J. G. (2001). Autonomous underwater vehicle homing/docking via electromagnetic guidance. IEEE J. Ocean. Eng. 26, 515–521. 10.1109/48.972086

[B15] Fossen T. I. Pettersen K. Y. Galeazzi R. (2015). Line-of-sight path following for dubins paths with adaptive sideslip compensation of drift forces. IEEE Trans. Contr. Syst. Technol. 23, 820–827. 10.1109/TCST.2014.2338354

[B16] Gross M. G. (1977). Oceanography: a view of the earth. Englewood Cliffs, NJ: Prentice-Hall, Inc.

[B17] Hartley R. I. (1999). Theory and practice of projective rectification. Int. J. Comput. Vis. 35, 115–127. 10.1023/A:1008115206617

[B18] Hine R. Willcox S. Hine G. Richardson T. (2009). The wave glider: a wave-powered autonomous marine vehicle. In OCEANS 2009, Biloxi, MS, United States, 26–29 October 2009. 1–6. 10.23919/OCEANS.2009.5422129

[B19] Hobson B. W. McEwen R. S. Erickson J. Hoover T. McBride L. Shane F. (2007). The development and Ocean testing of an AUV docking station for a 21" AUV. In OCEANS 2007, Vancouver, BC, Canada, October 2007. 1–6. 10.1109/OCEANS.2007.4449318

[B20] Jones C. P. (2012). Slocum glider persistent oceanography. IEEE/OES Autonomous Underwater Vehicles (AUV) 2012, Southampton, United Kingdom, 24-27 September 2012. 1–6. 10.1109/AUV.2012.6380738

[B21] Kanungo T. Mount D. M. Netanyahu N. S. Piatko C. D. Silverman R. Wu A. Y. (2002). An efficient k-means clustering algorithm: analysis and implementation. IEEE Trans. Pattern Anal. Mach. Intell. 24, 881–892. 10.1109/TPAMI.2002.1017616

[B22] Lambiotte J. C. Coulson R. Smith S. M. An E. (2002). Results from mechanical docking tests of a morpheus class auv with a dock designed for an oex class auv. In OCEANS`02 MTS/IEEE, Biloxi, MI, United States, 29-31 October 2002, 1, 260–265. 10.1109/OCEANS.2002.1193281

[B23] Lewis R. Bose N. Lewis S. King P. Walker D. Devillers R. (2016). Merlin—a decade of large auv experience at memorial university of newfoundland. In 2016 IEEE/OES Autonomous Underwater Vehicles (AUV), Tokyo, Japan, 6-9 November 2016. 222–229. 10.1109/AUV.2016.7778675

[B24] Li B. Page B. R. Hoffman J. Moridian B. Mahmoudian N. (2019). Rendezvous planning for multiple auvs with mobile charging stations in dynamic currents. IEEE Robot. Autom. Lett. 4, 1653–1660. 10.1109/LRA.2019.2896899

[B25] Li D. Zhang T. Yang C. (2016). Terminal underwater docking of an autonomous underwater vehicle using one camera and one light. Mar Technol Soc J 50, 58–68. 10.4031/MTSJ.50.6.6

[B26] Lu F. Zhang H. Mi C. (2018). A two-plate capacitive wireless power transfer system for electric vehicle charging applications. IEEE Trans. Power Electron. 33, 964â€“969. 10.1109/TPEL.2017.2735365

[B27] Ma Z. Wang Y. Wang S. Yang Y. (2016). Ocean thermal energy harvesting with phase change material for underwater glider. Appl. Energy 178, 557–566. 10.1016/j.apenergy.2016.06.078

[B28] Manley J. Willcox S. (2010). The wave glider: a persistent platform for ocean science. In OCEANSâ€™10 IEEE SYDNEY, Sydney, Australia, 24-27 May 2010. 1–5. 10.1109/OCEANSSYD.2010.5603614

[B29] Mateos L. A. Wang W. Gheneti B. Duarte F. Ratti C. Rus D. (2019). Autonomous latching system for robotic boats. 2019 International Conference on Robotics and Automation (ICRA), Montreal, QC, Canada, 20-24 May 2019, 7933–7939. 10.1109/ICRA.2019.8793525

[B30] Matsuda T. Maki T. Masuda K. Sakamaki T. (2019). Resident autonomous underwater vehicle: underwater system for prolonged and continuous monitoring based at a seafloor station. Robot. Autonom. Syst. 120, 103231. 10.1016/j.robot.2019.07.001

[B31] McEwen R. S. Hobson B. W. McBride L. Bellingham J. G. (2008). Docking control system for a 54-cm-diameter (21-in) auv. IEEE J. Ocean. Eng. 33, 550–562. 10.1109/JOE.2008.2005348

[B32] Meng L. Lin Y. Gu H. Bai G. Su T.-C. (2019). Study on dynamic characteristics analysis of underwater dynamic docking device. Ocean Eng. 180, 1–9. 10.1016/j.oceaneng.2019.03.033

[B33] Meng L. Lin Y. Gu H. Su T.-C. (2018). Study on the mechanics characteristics of an underwater towing system for recycling an autonomous underwater vehicle (auv). Appl. Ocean Res. 79, 123–133. 10.1016/j.apor.2018.07.014

[B34] Morgado M. Oliveira P. Silvestre C. Vasconcelos J. F. (2014). Embedded vehicle dynamics aiding for usbl/ins underwater navigation system. IEEE Trans. Contr. Syst. Technol. 22, 322–330. 10.1109/TCST.2013.2245133

[B35] Nad D. Ribeiro M. Silva H. Ribeiro J. Abreu P. Miskovic N. (2016). Cooperative surface/underwater navigation for auv path following missions. IFAC-PapersOnLine 49, 355–360. 10.1016/j.ifacol.2016.10.430

[B36] Naglak J. E. Page B. R. Mahmoudian N. (2018). Backseat control of sandshark auv using ros on raspberrypi*. In OCEANS 2018 MTS/IEEE Charleston, Charleston, SC, United States, 22-25 October 2018. 10.1109/OCEANS.2018.8604630

[B37] Neasham J. A. Goodfellow G. Sharphouse R. (2015). Development of the “Seatrac” miniature acoustic modem and USBL positioning units for subsea robotics and diver applications. In OCEANS 2015, Genova, Italy, 18-21 May 2015. 1–8. 10.1109/OCEANS-Genova.2015.7271578

[B38] Page B. R. Mahmoudian N. (2020). Simulation-driven optimization of underwater docking station design. IEEE J. Ocean. Eng. 45, 404–413. 10.1109/JOE.2018.2885200

[B39] Page B. R. Ziaeefard S. Pinar A. J. Mahmoudian N. (2017). Highly maneuverable low-cost underwater glider: design and development. IEEE Robot. Autom. Lett. 2, 344–349. 10.1109/LRA.2016.2617206

[B40] Painter H. Flynn J. (2006). Current and future wet-mate connector technology developments for scientific seabed observatory applications. In OCEANS 2006 MTS/IEEE, Boston, MA, United States, 18-21 September 2006, 1–6. 10.1109/OCEANS.2006.306829

[B41] Palomeras N. PeÃ±alver A. Massot-Campos M. Vallicrosa G. Negre P. L. FernÃ¡ndez J. J. (2014). I-auv docking and intervention in a subsea panel. In 2014 IEEE/RSJ International Conference on Intelligent Robots and Systems, Chicago, IL, United States, 14-18 September 2014. 2279–2285. 10.1109/IROS.2014.6942870

[B42] Pearson D. An E. Dhanak M. von Ellenrieder K. Beaujean P. (2014). High-level fuzzy logic guidance system for an unmanned surface vehicle (usv) tasked to perform autonomous launch and recovery (alr) of an autonomous underwater vehicle (auv). *2014* IEEE/OES autonomous underwater vehicles (AUV), Oxford, MS, United States, 6-9 October 2014, 1–15. 10.1109/AUV.2014.7054403

[B43] Pyle D. Granger R. Geoghegan B. Lindman R. Smith J. (2012). Leveraging a large uuv platform with a docking station to enable forward basing and persistence for light weight auvs. In 2012 Oceans, Hampton Roads, VA, United States, 14-19 October 2012. 1–8. 10.1109/OCEANS.2012.6404932

[B44] Sans-Muntadas A. Pettersen K. Y. Brekke E. Henriksen V. F. (2016). A hybrid approach to underwater docking of auvs with cross-current. OCEANS 2016 MTS/IEEE Monterey, Monterey, CA, United Sttaes, 19-23 September 2016. 1–7. 10.1109/OCEANS.2016.7761213

[B45] Sarda E. I. Dhanak M. R. (2019). Launch and recovery of an autonomous underwater vehicle from a station-keeping unmanned surface vehicle. IEEE J. Ocean. Eng. 44, 290–310. 10.1109/JOE.2018.2867988

[B46] Scaramuzza D. Martinelli A. Siegwart R. (2006). A toolbox for easily calibrating omnidirectional cameras. In 2006 IEEE/RSJ International Conference on Intelligent Robots and Systems, Beijing, China, 9-15 October 2006. 5695–5701. 10.1109/IROS.2006.282372

[B47] Scibilia F. Jã¸rgensen U. Skjetne R. (2012). Auv guidance system for dynamic trajectory generation. IFAC Proceedings Volumes 45, 198–203. 10.3182/20120410-3-PT-4028.00033

[B48] Sherman J. Davis R. E. Owens W. B. Valdes J. (2001). The autonomous underwater glider "Spray". IEEE J. Ocean. Eng. 26, 437–446. 10.1109/48.972076

[B49] Shkel A. M. Lumelsky V. (2001). Classification of the dubins set. Robot. Autonom. Syst. 34, 179–202. 10.1016/s0921-8890(00)00127-5

[B50] Silverman M. C. Nies D. Jung B. Sukhatme G. S. (2002). Staying alive: a docking station for autonomous robot recharging. In Proceedings 2002 IEEE International Conference on Robotics and Automation (Cat. No.02CH37292), Washington, DC, United States, 11-15 May 2002. 1, 1050–1055. 10.1109/ROBOT.2002.1013494

[B51] Singh H. Bellingham J. G. Hover F. Lemer S. Moran B. A. von der Heydt K. (2001). Docking for an autonomous ocean sampling network. IEEE J. Ocean. Eng. 26, 498–514. 10.1109/48.972084

[B52] Stielau O. H. Covic G. A. (2000). Design of loosely coupled inductive power transfer systems. PowerCon 2000. 2000 International Conference on Power System Technology. Proceedings (Cat. No.00EX409), Perth, WA, Australia, 4-7 December 2000, 1, 85–90. 10.1109/ICPST.2000.900036

[B53] Stokey R. Allen B. Austin T. Goldsborough R. Forrester N. Purcell M. (2001). Enabling technologies for remus docking: an integral component of an autonomous ocean-sampling network. IEEE J. Ocean. Eng. 26, 487–497. 10.1109/48.972082

[B54] Teo K. An E. Beaujean P.-P. J. (2012). A robust fuzzy autonomous underwater vehicle (auv) docking approach for unknown current disturbances. IEEE J. Ocean. Eng. 37, 143–155. 10.1109/JOE.2011.2180058

[B55] Webb D. C. Simonetti P. J. Jones C. P. (2001). Slocum: an underwater glider propelled by environmental energy. IEEE J. Ocean. Eng. 26, 447–452. 10.1109/48.972077

[B56] Wiggins S. Manley J. Brager E. Woolhiser B. (2010). Monitoring marine mammal acoustics using wave glider. OCEANS 2010 MTS/IEEE SEATTLE, Seattle, WA, United States, 20-23 September 2010, 1–4. 10.1109/OCEANS.2010.5664537

[B57] Wu L. Li Y. Liu K. Wang S. Ai X. Li S. (2019). A physics-based simulation for AUV underwater docking using the MHDG method and a discretized propeller. Ocean Eng. 187, 106081. 10.1016/j.oceaneng.2019.05.063

[B58] Xie W. Ma B. Fernando T. Iu H. H.-C. (2018). A new formation control of multiple underactuated surface vessels. Int. J. Contr. 91, 1011–1022. 10.1080/00207179.2017.1303849

[B59] Yan Z. Gong P. Zhang W. Li Z. Teng Y. (2019). Autonomous underwater vehicle vision guided docking experiments based on l-shaped light array. IEEE Access 7, 72567–72576. 10.1109/ACCESS.2019.2917791

[B60] Yang C. Peng S. Fan S. Zhang S. Wang P. Chen Y. (2016). Study on docking guidance algorithm for hybrid underwater glider in currents. Ocean Eng. 125, 170–181. 10.1016/j.oceaneng.2016.08.002

[B61] Yazdani A. M. Sammut K. Yakimenko O. Lammas A. (2020). A survey of underwater docking guidance systems. Robot. Autonom. Syst. 124, 103382. 10.1016/j.robot.2019.103382

[B62] Yuen H. Princen J. Illingworth J. Kittler J. (1990). Comparative study of hough transform methods for circle finding. Image Vis Comput. 8, 71–77. 10.1016/0262-8856(90)90059-E

[B63] Zhang L. Li Y. Pan G. Zhang Y. Li S. (2019). Terminal stage guidance method for underwater moving rendezvous and docking based on monocular vision. In OCEANS 2019—Marseille, Marseille, France, 17-20 June 2019. 1–6. 10.1109/OCEANSE.2019.8867192

[B64] Zhang S. Yu J. Zhang A. Zhang F. (2013). Spiraling motion of underwater gliders: modeling, analysis, and experimental results. Ocean Eng. 60, 1–13. 10.1016/j.oceaneng.2012.12.023

[B65] Zhang W. Wong S.-C. Tse C. K. Chen Q. (2014). Design for efficiency optimization and voltage controllability of series-series compensated inductive power transfer systems. IEEE Trans. Power Electron. 29, 191–200 10.1109/tpel.2013.2249112

